# Uveal coloboma: about 3 cases at the University Teaching Hospital, Yaounde, Cameroon

**DOI:** 10.11604/pamj.2016.24.201.9770

**Published:** 2016-07-08

**Authors:** Kagmeni Giles, Cheuteu Raoul, Bilong Yannick, Wiedemann Peter

**Affiliations:** 1 University Teaching Hospital Yaounde (UTHY), Cameroon; 2 University of Yaoundé I, Faculty of Medicine and Biomedical Sciences, Cameroon; 3 Eye Hospital of Leipzig University, Germany

**Keywords:** Uveal coloboma, congenital eye malformation, retinal detechment

## Abstract

Uveal coloboma is a rare eye malformation caused by failure of the optic fissure to close during the fifth to seventh weeks of foetal life. The risk of retinal detachment increases with age in colobomatous eyes. Preventive measures such as early detection of the retinal break, prophylactic laser photocoagulation along the coloboma margin, confer a significant benefit in reducing this risk of retinal detachment. Difficulties linked to the diagnosis and management of uveal colobomas in developing countries setting are presented in this study.

## Introduction

Uveal colobomas are a congenital eye disease caused by the defective closure of the embryonic optic fissure during the fifth to seventh weeks of foetal life [1]. Its prevalence is around 0.5 to 0.7 per 10,000 births, making it a relatively rare condition [2]. The effect of uveal colobomas on visual function varies according to the size and the location in the eye. With the aid of 3 cases we present the clinical characteristics and the difficulties involved in the management of uveal colobomas in our IONS.

## Patient and observation

**Figure 1:**
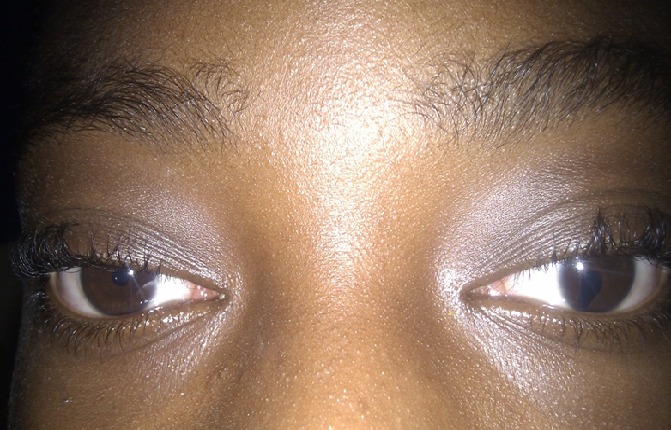
Iris coloboma of the left eye showing the classic keyhole-shaped defect

**Patient 1:** A 14-year-old female was referred from the paediatrics service with severe bitemporal headaches aggravated by reading. Her uncorrected distance visual acuity was 1.0 in both eyes; slit-lamp examination was normal in the right eye and revealed in the left eye an inferior nasal iris notch giving an appearance of the classic keyhole-shaped defect (Figure 1). Fundus examination of the right eye was normal. On the left eye the choroid was absent, and there was atresia on the inferonasal retina, exposing sclera. The macula was not involved, nor was the retina periphery in 3-mirror goniolens examinations. Cycloplegy refraction revealed a hypermetropia of + 2.5. A diagnosis of uveal coloboma was made. Optical lenses for hypermetropia were prescribed and the patient was addressed to paediatrician for systemic associated abnormality check.

**Patient 2:** A 60-year-old woman presented with a painful, swelling lower lid of the left eye, for which she used self-prescribed eyedrops for 3 days. Corrected distant visual acuity was 1.0 in both eyes. Near visual acuity was at Perinaud 2. Slit-lamp examination revealed a progredient, age-related cataract in both eyes. In the left eye, there was an inflammation on the root of an eyelash on the lower eyelid, and the iris presented a keyhole-shaped defect lesion at 6-7 o’clock position. Dilated fundus examination was normal in both eyes. The diagnosis of a hordeolum associated with unilateral iris coloboma was made. Local treatment was administered, consisting of an antibiotic and an anti-inflammatory.

**Figure 2:**
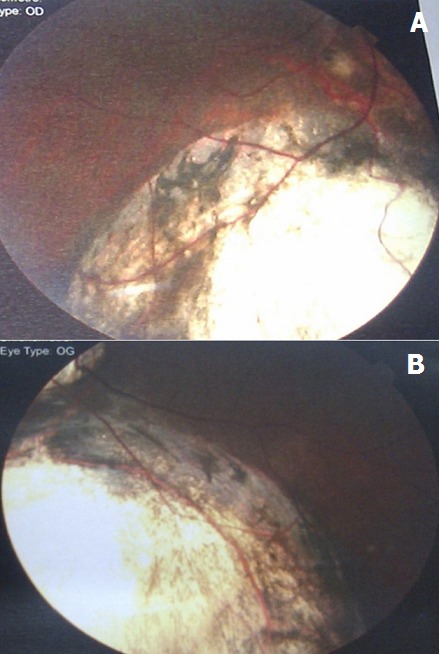
Bilateral chorio retinal coloboma showing atresia on the inferonasal retina, exposing sclera without macula involvement. A) right eye; B) left eye

**Patient 3:** A 12-year-old student consulted because of a decrease in visual acuity despite having worn corrective lenses for 3 years. Her history showed nothing in particular. Distant visual acuity was 0.1 in both eyes. Slit-lamp examination of both eyes indicated the presence of iris coloboma at the 6 or 7 o’clock position. Fundus examination revealed myopic conus and a chorioretinal coloboma in the infero nasal quadrant without macula involvement (Figure 2). After refraction under cyclopegia, the best corrected visual acuity was 0.5 in the right eye (sphere -6 combined to cylinder -2.50 at 0°) and 0.3 in the left eye (sphere -7 combined to cylinder -2.00 at 5°). The patient was prescribed corrective lenses and was referred for systemic associated abnormalities exclusion.

## Discussion

Uveal colobomas are a congenital malformation that is secondary to a failure in the closure of the embryonic optic fissure [1]. First described in the iris in 1673 by Bartholin the Younger [2], iris coloboma represents about 2% of ocular malformations [3]. Our 3 patients were all female. Uhumwando et al. reported a male predominance in a series of 198 patients [4]. An iris coloboma can occur in association with a retinal coloboma and a choroid coloboma (as in the case of patients 1 and 3) or an affection of the optic papilla. The visual prognosis in eyes with coloboma is highly variable and depends on macular or optic nerve involvement, or globe disorganisation. For 2 of our patients the best corrected visual acuity was 10/10 in both eyes (patients 1 and 2). The decrease in visual acuity observed inpatient 3 was attributed to anisometropy amblyopia caused probably by the chorio retinal coloboma. Choroidal neovascularisation (CNV) has been identified as cause of vision loss in 3 cases of coloboma of choroid [5]. Chorioretinal colobomas are responsible for 0.5% of all retina detachments in young people [6]. In one observational case series of consecutive patients aged 0-15 years with chorioretinal coloboma, a prevalence of retinal detachment of 17.6% was reported [4]. Tears, which are the main cause of retinal detachment, are usually located intracolobomally in younger patients or extracolobomally in older subjects. Multiple and peripheral tears should always be checked. OCT is the more reliable means in the diagnosis of intracolobomal retinal breaks or detachments. In our seriesfundus examination with an indirect ophthalmoscope did not reveal any retina break in two of our patients who presented a chorioretinal coloboma. Although they presented a potential risk of retina detachment, they did not received a prophylactic laser photocoagulation along the coloboma margin as this confers a significant benefit in reducing this risk of retina detachment [4]. Nevertheless, they were educated on the signs and symptoms of retinal detachment.

Uveal colobomas can exist in isolation or in association with other ocular abnormalities. The most common associated ophthalmic abnormalities include strabismus, microphthalmia and microcornea, nystagmus, myopia and posterior staphyloma. The presence of microcornea and microphthalmia indicates a poor visual prognosis [7]. No associated ocular abnormalities were seen in patients 1 and 2. A severe myopia found in patient 3 was likely due to the coloboma. Uveal colobomas were found to be associated with many genetic syndromes such as Goltz syndrome [6] and Ascher's syndrome [8]. Maumenee et al. found that 27% of patients with colobomas also presented with a systemic abnormality of varying severity [9]. Our patients did not present any clinically systemic abnormality. However, Nancy et al. strongly recommended a physical examination by a clinician that may guide further evaluation, such as echocardiography, an audiology assessment, kidney ultrasound or spine X-ray, for all patients presenting with apparently isolated uveal colobomas [10]. Our patients did not undergo OCT examination because of our limited technical equipment. This constitutes a limitation of our study.

## Conclusion

Although uveal colobomas are a rare entity, they are present in our milieu. It was discovered by chance in our 3 patients, who were all female. No case of systemic malformation was found. However, these patients need to be followed because they all run the risk of developing retinal detachments.
